# Influence of External Thermal Conditions on Temperature–Humidity Parameters of Indoor Air in a Czech Dairy Farm during the Summer

**DOI:** 10.3390/ani12151895

**Published:** 2022-07-25

**Authors:** Pavel Kic

**Affiliations:** Department of Technological Equipment of Buildings, Faculty of Engineering, Czech University of Life Sciences Prague, 16521 Prague, Czech Republic; kic@tf.czu.cz

**Keywords:** calf hutches, calf shelters, dairy cows, heat stress, housing, milking parlor, temperature attenuation, temperature waveform models

## Abstract

**Simple Summary:**

The creation and provision of a suitable indoor environment for animals in dairy farms has become increasingly important in recent years, especially in the summer. Greater attention is paid mainly to lactating dairy cows. This research shows that great attention should be paid not only to cowsheds for lactating dairy cows but also to the housing facilities for other categories of cattle kept on farms. In this article, the basic parameters of the thermal state of the environment during the summer period are assessed regarding the housing facilities. The analysis shows that more attention needs to be paid to the housing of calves. In the facilities for calves, the values of the thermal state of the environment were at an extremely dangerous level, especially the high air temperatures, which exceed the recommended limit values.

**Abstract:**

The aim of this article is to show the relationship between external thermal conditions and the quality of the indoor environment on a dairy farm during the summer. The measurements were carried out on a large dairy farm of Holstein cattle situated in the Czech Republic. The research included the measurement of the cowshed for 440 lactating cows, a milking parlor, a maternity cowshed, a cowshed for dry cows, 69 individual calf hutches, and three outdoor group shelters for calves. The results of the registration measurements of the thermal state parameters outside and inside the buildings were analyzed. The critical and dangerous situations were especially regarding the calves. The highest temperature in the calf hutches was 48 °C with the value of THI_max_ = 90.1, while in the calf group shelters it was 46.9 °C with the value of THI_max_ = 89.4. The research results showed that not only the critical values of temperature and the temperature–humidity index that affect the housed animals are important but also the duration for which the animals are exposed to heat stress. The massive masonry constructions of the milking parlor and also of the cowshed for dry cows dampened the temperature rise in these barns, with good values for the attenuation coefficient.

## 1. Introduction

According to research in recent years, the incidence and intensity of heat waves in Europe are expected to increase with climate change [1,2]. The Environmental Report of the Czech Republic shows that the number of warm days is increasing [3]. The increase in the average air temperature in the period 1961–2020 is obvious in all seasons. The number of days with extreme heat in the Czech Republic has doubled since the 1980s [4]. The annual number of tropical days, i.e., those when the temperature exceeds 30 °C, has more than doubled in 30 years. It is estimated that this trend will continue for at least the next few decades. These high temperatures can have serious effects on farm animals.

Agriculture, including livestock production, also has a share of the impact on climate change [5]. The influence of livestock as a farming sector on the ecosystem is very large [6]. Reducing animal numbers whilst increasing the average yield per animal has great potential to decrease greenhouse gases (GHGs) per unit product [7]. However, this requires the creation of breeding conditions, including suitable housing, to increase the breeding production of farm animals.

Hot weather can strongly affect animal bioenergetics, with adverse effects on the performance and wellbeing of livestock. Only if the values of the environment are at the optimal level (usually recommended temperature within a range of −0.5 to 20.0 °C and 40–80% relative humidity), the dairy cows are in a state of well-being [8,9,10,11]. The results of some research studies revealed that even mild heat stress reduced the feed intake and milk yield of dairy cows [12,13]. The influence of temperature conditions in the hot tropical climate on milk production is very strong and causes serious problems, especially for dairy cows with high milk yield [14]. Due to intense metabolic processes, high-producing dairy cows are highly vulnerable to the effects of heat stress [15]. Heat stress has great economic impacts on animal production [16].

Several research studies evaluated the combined influence of air temperature and humidity expressed in the temperature–humidity index (THI) as a suitable criterion for the evaluation of the microclimate [17,18,19,20,21,22]. The authors state certain thresholds in their publications. THI values suggest that within the normal range up to 70 (THI ˂ 70) cattle show optimal performance (thermoneutral range). Mild heat stress occurs when 70 ≤ THI ˂ 74, heat stress occurs when 74 ≤ THI ˂ 77, and severe heat stress occurs when THI ≥ 77. Dairy cattle are beginning to be stressed when the THI exceeds 72 [18], while critical THI values occur over 78.

According to [11,23], THI above 72 causes heat stress. THI increased from 67 to 78 decreased milk production by 21% and dry matter intake by 9.6%. Heat stress is mainly influenced by high air temperatures exceeding the thermoneutral zone, usually reported in the range of −0.5 to 20 °C and at a relative humidity in the range of 60% to 80%. The recommended upper limit temperature at which Holstein cattle maintain a constant body temperature is 25 °C. However, the problem occurs when the relative humidity is high, then the milk yield decreases [24]. Expressed as a THI, a decrease in milk yield occurs at a THI higher than 77. Based on many studies, using the THI as the thermal environment indicator, the critical values for minimum, mean, and maximum THI are 64, 72, and 76, respectively [25].

According to [9,26,27], the maximum daily air temperature has a great effect on reducing milk yield. During warming, to a critical range of THI of 70–72, performance of dairy cattle is inhibited [26,27], and deterioration in milk yield occurs at THI from 72 to 78. A critical THI to dairy cows and severe deterioration in milk yield occur from 78 to 82. THI values above 82 is dangerous, and deaths may occur. According to [28], the risk of death in dairy cows starts to increase when the maximum daily THI is above 80.

An upper critical temperature of 25 °C is recommended for calves [29] and, according to [30], the maximum permissible temperature for calves is 28 °C, if the maximum relative humidity is between 50% and 70%. According to research [31], calves tolerate high temperatures and heat stress better than adult cattle. Heat stress in calves occurs at THI values from approximately 78 to 88.

The microclimatic conditions were influenced both by the season and by the influence of the farm (holding area, milking parlor, housing area) [32]. A suitably architecturally designed building should contribute to reducing energy consumption in buildings, while creating a comfortable environment [33]. From this point of view, it is important to design suitable ventilation for milking parlors, so that dairy cows are not exposed to difficult conditions and heat stress during milking [34].

Modern cowsheds in countries with intensive dairy farming are most often uninsulated and equipped with natural ventilation. The intensity of ventilation is influenced by the temperature of the indoor and outdoor air temperature, the size, the location of the supply and exhaust openings for air exchange, and, last but not least, the size and direction of the wind [35,36].

Air cooling has led to significant progress in improving the thermal comfort of the lactating dairy cow [37]. Currently, air movement (fans), wetting (soaking) the cow’s body surface, high-pressure mist (evaporation) to cool the air in the cows’ environment, and facilities designed to minimize the transfer of solar radiation are used for heat abatement. A review [38] provides a comprehensive overview of the importance and development of views on the assessment of heat stress in dairy cattle. The article also includes the economic impacts of heat stress on milk yield; fans and sprinklers are a good investment for cow cooling. From the point of view of practical help for farmers, the overall conclusion is interesting: fans plus misters are still the most cost-effective heat-stress mitigation approach, even in temperate climates. The ability to provide such mitigation solutions depends on many factors, and with increased monetary pressures on the agricultural sector from climate change, mitigation techniques require careful consideration of efficacy, energy use, and capital outlay.

In countries with colder climates and longer winters (e.g., some areas of northern Europe), not only winter temperatures but also average summer temperatures are lower [39,40,41], and, therefore, the construction of animal houses has for many years been based on the requirement of protecting animals from the cold in winter. The situation is similar in the countries of Central Europe. These buildings are gradually being modernized and used. Research should examine how greater heat capacity and insulation of buildings will help dampen heat gains in the summer.

The methods of so-called passive air conditioning use the surface-reflective properties of some materials used for walls and roofs to reduce excessive heat loads by radiation. The use may also be important for the construction of housing systems for calves, e.g., calf hutches [42,43,44]. Properly designed covering of calf hutches can contribute to the improvement of the indoor environment, even in winter, by reducing heat losses [45].

Another option for passive air conditioning is to use the heat-storage capabilities of massive wall and ceiling structures, which were previously often used for the construction of cowsheds. Part of the heat transferred to the building by radiation and convection is stored in the massive structure of the building, and the highest internal temperature is, thus, dampened and its peak is shifted to a later time. This not only reduces the highest indoor air temperatures overall, but also shortens the duration of heat stress for housed animals [46].

New cowsheds are usually designed as uninsulated and are ventilated naturally with air extraction through roof ridges and air supply through net walls and other open areas. The roof only protects against rain, snow, and sunlight. Therefore, less attention is paid in the scientific literature to the issue of the heat capacity of cowsheds and the related effects.

In other types of buildings, the thermal capacity of the building is becoming an increasingly important factor contributing to the creation of the internal thermal state of the microclimate [46,47,48]. Experience from countries with warm climates, such as Italy, shows that the use of suitable materials, in some cases even very environmentally friendly materials, can significantly improve the temperature conditions in buildings in summer [48,49]. It can be assumed that similar applications would also help buildings for livestock.

Finding shade is the natural behavior of cattle. Access to shade prevented the decrease in milk yield that was observed in cows without access to shade [50]. It has been well-established that the provision of shade is an advantageous heat-load-alleviation tool for lactating dairy cows [9]. Recently, more and more attention has been paid to the fact that in some countries the agriculture, including animal production and cattle breeding, is industrialized and, e.g., cows are reared indoors most of the year, as it is routine practice to separate the calf from the cow within 24 h of calving. Farm industrialization aiming at increasing animal production by reducing space and resources has negatively affected animal welfare. Properly designed cowshed and environmental technology equipment are a means of maintaining the comfort parameters of the suitable microclimate [51]. The economic impact of some environmentally friendly technologies was assessed on a dairy cattle farm located in southern Minas Gerais state (Brazil) [52]. Environmentally friendly technologies contributing to cost reduction can be alternatives for sustainability in dairy farming, especially concerning environmental and economic aspects of production systems.

Using a suitable technique (e.g., air cooling by a fogging system), we can compensate for some deviations in the parameters that are caused by the poor construction of the building or the age of the building. However, even in the case of a cowshed, the principles of passive air conditioning can also be used. For example, the choice of suitable building materials, suitable shape of the roof, suitable location and size of inlet and outlet openings for natural ventilation, etc. In a study [53], a parametric analysis of several traditional or innovative passive solutions for building envelope of livestock housing is carried out, to achieve the thermal comfort of animals without active energy systems.

This article aims to show the relationship between external thermal conditions and the quality of the indoor environment in the basic sections of a large-capacity dairy farm in the summer.

## 2. Materials and Methods

### 2.1. Description of the Farm

This research work and measurements were carried out on a dairy farm situated in the central part of the Czech Republic (50°08′28.7″ N 13°52′30.9″ E, mean elevation of 412 m above sea level). The dairy farm, with parameters that correspond to current farms in the Czech Republic, specializes in the breeding of Holstein cattle with an average yield of 8310 kg of milk per lactation. It is situated in conditions of temperate Central European climate with cold winters, where the cows have to be protected from the wind and precipitation, but rather a hot summer, where they have to be protected from the sun and heat.

The research included the measurement and evaluation of buildings: a dairy production cowshed for lactating cows (LC) with a capacity of 440 dairy cows, a milking parlor (MP), a special maternity cowshed for calving (MC), a cowshed for dry cows (DC), 69 individual outdoor hutches for calves in the first phase of breeding (CaH), and three outdoor group shelters for older calves from 2 to 4 months of age (CaS).

**Figure 1:**
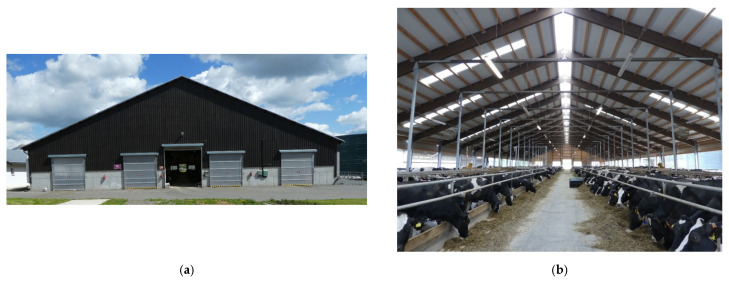
The cowshed for lactating cows (LC): (**a**) the front view of the cowshed; (**b**) the interior of the cowshed.

The main building for lactating cows (LC) (Figure 1) is a new modern cowshed for loose housing of lactating cows with dimensions of 110 m × 34.5 m, which has a capacity of 440 dairy cows (408 cows housed at the time of measurement). The LC is a semi-closed non-insulated building with comfort cubicles covered by separated dried manure solids as bedding. Longitudinal walls are made of woven fabric mesh and variable side curtains. The roofing is made of PUR sandwich roof panels (polyurethane roof panels), with translucent strips (approx. 10% of the roof area). Five rolling doors are mounted in the transverse front and rear wall, four of which measure 3.5 m × 3.5 m. The gate in the middle of the barn measures 4 m × 4.2 m and is also used as a gate for the feeding corridor.

The cowshed for lactating cows has natural ventilation with a ventilation roof ridge slot. In the summer months, a system of 24 axial fans switched on automatically at 24 °C helps to improve convection cooling and remove air from the building.

**Figure 2:**
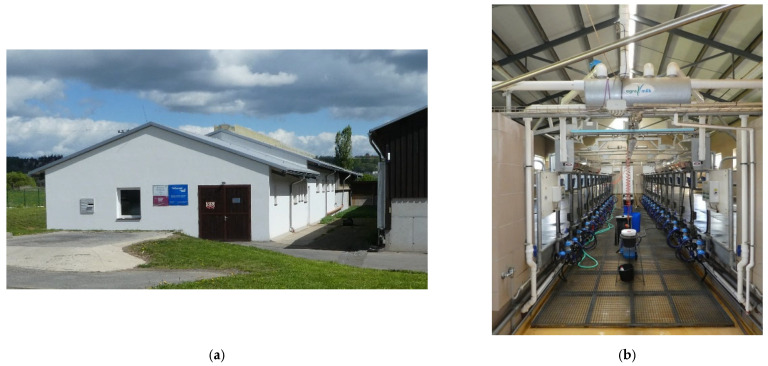
The herringbone milking parlor (MP): (**a**) exterior view; (**b**) the interior of the milking parlor.

The herringbone milking parlor (MP) is a modern brick construction that measures 66 m × 11 m (Figure 2). The wall thickness is 45 cm, made of perforated bricks for perimeter masonry. The roofing is made of PUR sandwich roof panels (Polyurethane roof panels). It has 2 × 12 milking stalls with rapid exit. The milking parlor is situated on the northwest side of the LC and is connected by a covered corridor.

**Figure 3:**
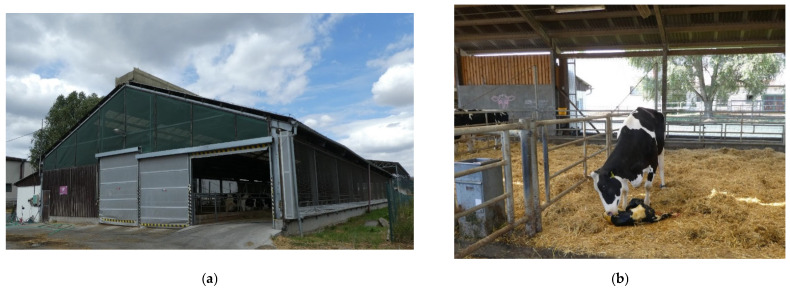
The maternity cowshed (MC): (**a**) exterior view; (**b**) the interior of the maternity cowshed.

The housing of dairy cows before and at the time of calving is in a maternity cowshed (MC), which is a modernized cowshed measuring 40 m × 16.6 m with an extension (milking parlor for maternity cows) measuring 9.3 m × 3.2 m. The maternity cowshed has free housing with straw bedding in nine maternity pens, with a total capacity of 27 housing places (Figure 3). It has corrugated fiber cement roofing, with translucent strips. Natural ventilation allows a ventilation ridge roof vent, a side wall made of woven fabric mesh and variable side curtains, and the rolling doors (3.5 m × 3.5 m), which are arranged at the entrance and exit of the feed corridor. In the summer months, 8 axial fans switched automatically at 24 °C helps to improve convection cooling.

**Figure 4:**
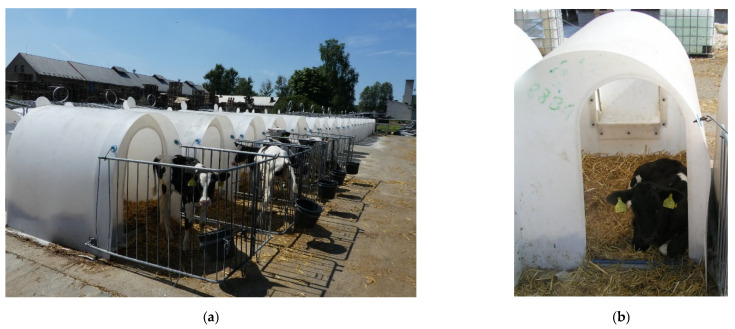
The calf hutches (CaH): (**a**) the group of calf hutches; (**b**) detail of a calf inside the hutch.

Individual outdoor hutches (CaH) (Figure 4) for housing calves in the first breeding period up to 2 months of age are made of white polyethylene. All the CaH have a length of 150 cm, a width of 112 cm, and a height of 135 cm. The floor is covered daily with new straw bedding.

**Figure 5:**
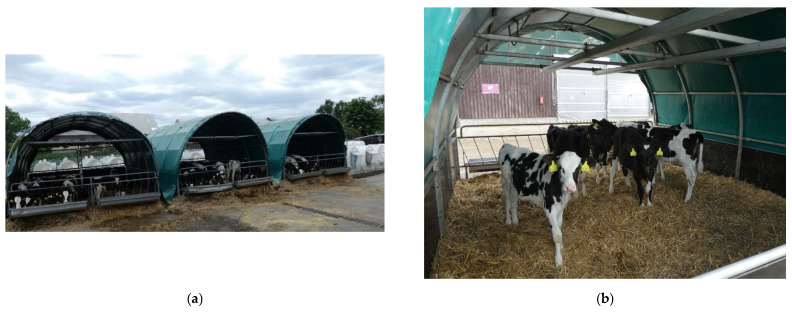
The calf shelters (CaS): (**a**) the group of calf shelters; (**b**) detail of calves inside the shelter.

Three outdoor group shelters (CaS) for older calves from 2 to 4 months of age have a lightweight construction measuring 5.5 m × 4.5 m. The frame is made of steel, and the green cover of the animal shelter is made of very durable PVC material. Usually, 6 to 10 calves are housed in each of them as needed (Figure 5). The floor is covered with deep straw bedding.

**Figure 6:**
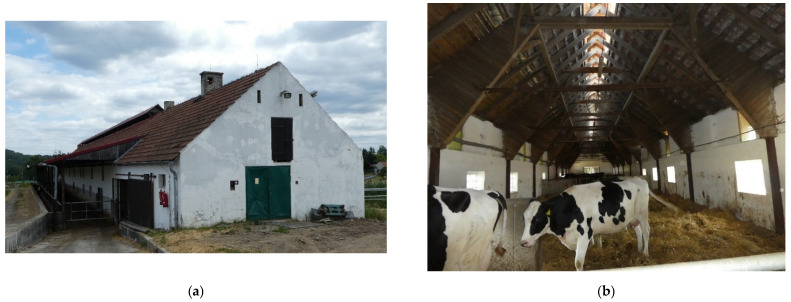
The cowshed for dry cows (DC): (**a**) exterior view; (**b**) the interior of the cowshed for dry cows.

The modernized cowshed for loose housing of dry cows (DC) is a massive brick structure measuring 45.7 m × 7 m. It is traditional solid brick construction, made of solid fired bricks with internal and external plaster, with thickness of 60 cm. The roof is covered only with traditional roof tiles without additional insulation and without ceiling. It is divided into an outdoor feeding passage and a rest area on deep litter, which is located inside the cowshed (Figure 6). Natural ventilation is enabled by a central roof ridge slot, window openings, and four fans. At the time of the measurement, 23 cows were housed in DC.

### 2.2. Data Acquisition and Processing

Air temperatures and relative humidity were measured by data loggers outside and inside the buildings, with registration at intervals of 15 min. For the purposes of this measurement, a total of eight data loggers were installed on the farm: two data loggers in the barn for lactating dairy cows, one data logger each in the other buildings. Two models of data loggers were used: four data loggers of type ZTH65 and four data loggers of type R3120. Manufacturer and supplier of dataloggers is company COMET SYSTEM, s.r.o. [54].

Parameters of ZTH65 are: temperature operative range −30 to +70 °C with accuracy ± 0.4 °C, resolution of temperature measurement results 0.1 °C; and operative range of relative humidity 5–95% with accuracy ± 2.5%, resolution of relative humidity measurement results 0.1%. Dimensions: 94 mm × 62 mm × 32 mm.

Parameters of data logger R3120 are: temperature operative range −30 to +80 °C with accuracy ± 0.4 °C, resolution of temperature measurement results 0.1 °C; operative range of relative humidity 5–95% with accuracy ±2.5%, resolution of relative humidity measurement results 0.1%. Dimensions: 93 mm × 64 mm × 27 mm.

The control, operation, and use of both types of data loggers is the same. Comet measuring instruments are first checked and prepared for measurement in laboratory conditions. After connecting the personal computer by IR adapter connection via USB, they are checked, and, if necessary, the settings of all parameters are uniformly adjusted (temperature and relative air humidity, date, time, storage interval of measured values, battery charge status). Starting and turning off the devices is done using a separate magnetic controller. The data are transferred by IR adapter connection to a PC via USB.

The data logger for outside temperature and humidity measurements was kept in a special weather station box to protect the sensors from sunshine, wind, and the surroundings. The data loggers for inside measurements were situated in representative positions of studied buildings near the living area of animals.

Due to the large length of the cowshed for lactating cows (LC), the two end parts of the cowshed LC SW (southwest) and LC NE (northeast) were measured and evaluated separately for this research. That enables study of the influence of the cowshed position not only in terms of construction and technology but also in terms of the influence of the cowshed position relative to the cardinal directions and the effects of sunlight and the direction of prevailing winds on the building and the indoor environment.

The effect of combinations of temperature and relative humidity is included in the THI, which can be calculated by several methods. According to one of the common methods of calculation, e.g., [18,19], the THI is determined by Equation (1).

(1) $$\mathrm{THI}\;=0.8\;\cdot {\;t}_{\mathrm{db}}+\frac{\left(t_{\mathrm{db}}-14.4\right)\;\cdot \;\mathrm{RH}}{100}+46.4\;$$

where THI—temperature–humidity index, –; t_db_—dry bulb temperature of the air, °C; and RH—relative humidity of air, %.

This article aims to show the effect of outdoor temperatures on the indoor environment during the hot summer period (HSP) of the current climatic conditions of Central Europe. The article, therefore, contains processed results of measurements of four days of measurement from 22 July to 25 July.

For a more detailed analysis of the influence of outdoor air temperature and other factors affecting the indoor thermal humidity in agricultural buildings, it is appropriate to analyze one selected day [55,56] and its modeling in applications on different species of livestock [57,58,59,60,61]. This method of analysis has proved its worth, especially in countries in Europe with higher air temperatures during the year, for example, in countries around the Mediterranean Sea. In some cases, it was possible to use airflow to improve natural ventilation and intensify the cooling effects [62,63,64].

For a more detailed analysis of the temperature–humidity conditions in the individual housing facilities, one representative hot summer day was also selected (24 July), and a model of the course of the air temperature during 24 h of this day was prepared.

Changes in the outside air temperature cause temperature fluctuations inside the housing, which is affected by the massiveness of the structure, the influence of the biological production of the sensible heat of animals, and the influence of ventilation intensity. In most theoretical considerations, it is assumed that the temperature changes are harmonic and have a sinusoidal course during the day [55,56].

To assess the influence of the building on the change in internal temperature, it is appropriate to introduce the technical term temperature attenuation, which can be expressed by the attenuation coefficient AC, according to Equation (2), which is used in a similar form [64,65,66].

(2) $$\;\mathrm{AC}=\frac{A_{i}}{A_{e}}$$

where AC—the attenuation coefficient; A_i_—the fluctuations of the internal air temperature, K; and A_e_—the fluctuations of external air temperature, K.

The fluctuations of the internal air temperature A_i_ are calculated, according to Equation (3), as half the difference of the maximum temperature of the internal air t_i,max_ minus the minimum indoor air temperature t_i,min_.

(3) $$A_{i}=\left(t_{i,\max}-t_{i,\min}\right)/2$$

where t_i,max_—the maximum temperature of the internal air, °C; t_i,min_—the minimum temperature of the internal air, °C.

The fluctuations of the external air temperature A_e_ is calculated, according to Equation (4), as half the difference of the maximum temperature of the external air t_e,max_ minus the minimum external air temperature t_e,min_.

(4) $$A_{e}=(t_{e,\max}-t_{e,\min})/2$$

where t_e,max_—the maximum temperature of the external air, °C; t_e,min_—the minimum temperature of the external air, °C.

It can be assumed that a more massive structure, which can accumulate thermal energy, can dampen fluctuations in the outside temperature and, thanks to heat accumulation and inertia, shift the temperature maximum by a certain time. The time shift of temperature maximum Ψ is the time difference between the maximum indoor air temperature and the maximum outdoor air temperature, calculated according to Equation (5).

(5) $$\Psi =\tau_{\mathrm{ti},\max}-\tau_{\mathrm{te},\max}$$

where Ψ—the time shift of temperature maximum, h; τ_ti,max_—the time of maximum internal air temperature, h; and τ_te,max_—the time of maximum external air temperature, h.

The acquired datasets were processed using MS Excel, and some of the results were verified by statistical software TIBCO SW Data Science Workbench Statistica Version 6 (*ANOVA* and *Tukey’s HSD (Honestly Significant Difference) test*).

## 3. Results

Average outdoor air temperature calculated for the measured 4 days of the hot summer period (HSP) was t = 24.8 ± 5.6 °C, average relative humidity RH = 51.0 ± 18.5%, and calculated average value THI = 70.5 ± 5.6. To assess the thermal–humidity conditions in the individual housing facilities, the results were processed (see Table 1). From the results in the table and the statistical evaluation of the measured datasets, certain differences between the individual housing objects are evident. From the results of measurements and statistical evaluation given in Table 1, it is clear that the calves shelter (CaS) and the calves hutch (CaH) have statistically significantly different temperatures and THI, compared to other housing facilities during the hot summer period (HSP).

**Figure 7:**
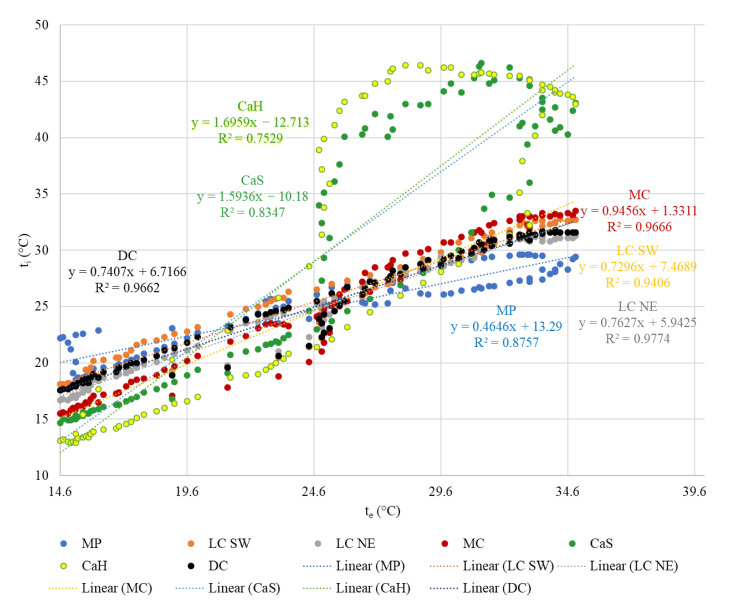
The dependence of the indoor air temperature on the outdoor air temperature during the hot summer day.

Figure 7 is a chart showing the dependence of the indoor air temperature on the outdoor air temperature during one hot summer day. The courses of temperature in the calves’ shelter (CaS) and the calves’ hutch (CaH) are very different in comparison with the other housing systems during the hot summer day.

The results of measured values during one hot summer day are in Figure 7, Table 2, Table 3 and Table 4. Table 2 shows the average temperatures t_1d_ ± SD °C, the average relative humidity RH_1d_ ± SD%, and the maximum temperatures t_1d max_.

From the point of view of heat stress, not only the level of critical value that affects the housed animals is important but also the duration for which the animals are exposed to the heat stress. Table 3 shows the calculated average values THI_1d_ ± SD, the time in hours, and the percentage of the examined period of HSD for which the significant limit values THI_1d_ ≥ 70, THI_1d_ ≥ 72, and THI_1d_ ≥ 78 are exceeded and the maximum values THI_1d max_ during one hot summer day are examined.

In most theoretical considerations, it is assumed that the temperature changes are harmonic and have a sinusoidal course within one day, i.e., 24 h. The results of the measured average air temperatures t_1d_ ± SD °C, attenuation coefficient AC_1d_, time shift of temperature maximum Ψ_1d_, model curve equations expressing the course of air temperature during 24 h of hot summer day, and average temperatures t_1dm_ ± SD °C calculated from the model waveforms are in Table 4.

**Figure 8:**
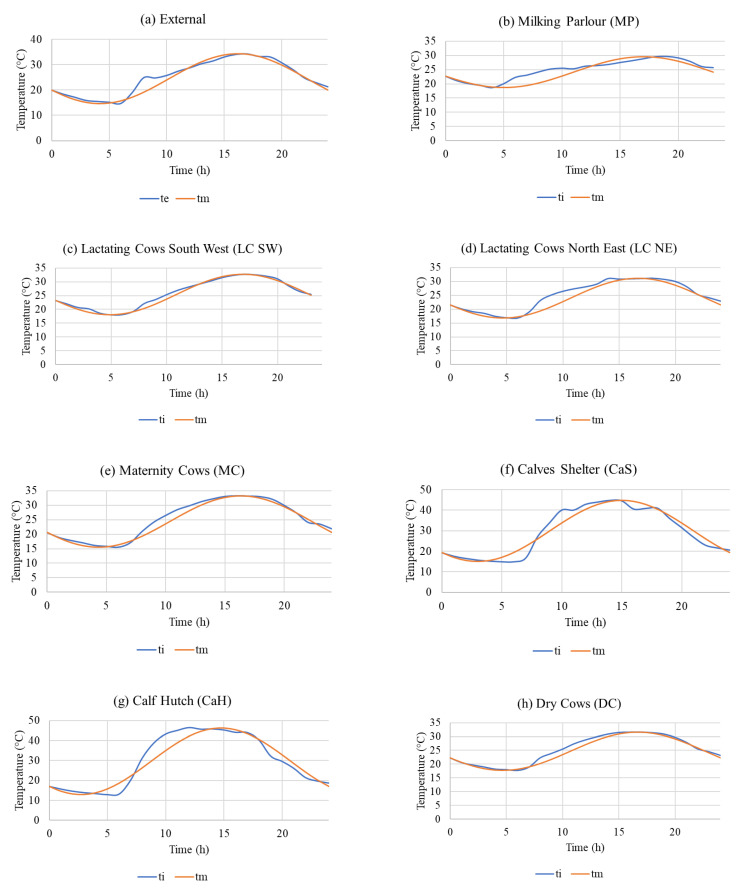
The course of the measured air temperature and the model sinusoidal course of the air temperature, according to the equations given in Table 4, during the 24 h of hot summer day in all examined objects: (**a**) External; (**b**) MP; (**c**) LC SW; (**d**) LC NE; (**e**) MC; (**f**) CaS; (**g**) CaH; (**h**) DC.

Figure 8 shows graphs expressing the course of the measured air temperature (results presented in Table 2) and the model sinusoidal course of the air temperature, according to the equations given in Table 4, during the 24 h of the hot summer day, for all examined objects. The compiled theoretical sinusoidal waveforms of air temperatures can, thus, be used in the discussion for comparison with the real waveforms. The analysis of the difference between the real and model waveforms will help to identify some of the causes of the problematic results found in the housing facilities.

For the measured values of air temperatures in the individual measured housing systems, the equations given in Table 4 best describe the sinusoidal course. According to the results in the table and from the statistical evaluation of the measured datasets, it follows that there is no statistically significant difference between the measured values in real objects and the values in the model curves, as shown in Figure 8.

## 4. Discussion

The results of this case study show some differences between the individual housing systems. The authors of [67] show that the average indoor temperature is in correlation with outdoors; this research also proved that the average air temperatures during the measured hot summer period in most housing buildings do not differ from the outdoor air temperature of 24.8 ± 5.5 °C. However, the average temperatures in the calf shelter (29.5 ± 10.2 °C) and in the calf hutch (30.3 ± 11.9 °C) exceeded the recommended [29] values of the critical temperature of 25 °C and the maximum permissible [30] temperature of 28 °C.

According to [17,18,19,20,21,22], maintaining a THI value below 70 means the absence of heat stress. The average values of THI during the hot summer period (Table 1) were higher than THI = 70 in all objects. According to [11], the accuracy of determining the beginning of heat stress depends on the production of cows. According to [26,27,42], critical values of THI ≥ 72 are when milk production is seriously affected. The data in Table 3 show that the length of time that the THI limit values were exceeded varied significantly for some buildings. Exceeding the THI value ≥ 72 were, in all cases, housing for dairy cows and calves for more than 12 h. The highest THI_max_ values exceeded THI = 76 in all buildings, which causes heat stress for dairy cows [17,18,19,20,21,22,23,24,25,26,27,28].

The highest THI_max_ values exceeded THI = 76 in all buildings, which corresponds to very high indoor air temperatures. The highest temperature t_max_ = 32 °C was exceeded in most buildings except the milking parlor. The critical situations in terms of the highest temperature were similar to [42,43,44] in the buildings for calves, in the calf shelter t_max_ = 46.9 °C, and in the calf hutch t_max_ = 48 °C. The critical values of THI_max_ also corresponded to these high temperatures, in the calf shelter THI_max_ = 89.4, and in the calf hutch THI_max_ = 90.1. These values exceeded the critical values [31].

The maximum measured air temperatures differed in most buildings during the observed hot summer period (Table 1). The maximum outdoor temperature (External) was t_max_ = 34.9 °C. The lowest maximum temperature was t_max_ = 29.6 °C in the milking parlor. The masonry construction of the milking parlor had a positive effect on the inside air temperature. This is evident from the analysis of the measurement and model of one hot day (Table 4 and Figure 8b). The massive masonry construction of the milking parlor, with greater heat storage that better dampened the temperature rise, which is also reported by [59,65], was reflected in the results of measurements and modeling similar to [64,65,66], for one day, with the values AC_1d_ = 0.55 and Ψ_1d_ = 0.75 h.

As a result of milking, similar to [34,36], the average relative humidity in the milking parlor (RH = 62.3 ± 13.5%) was the highest of all buildings (Table 1) in the measured hot summer period. The milking parlor also had a high average THI = 71.7 ± 3.4. The value of THI = 72, recommended by [11,18,23,25] as the limit, was exceeded for 16.8 h.

Thanks to the natural ventilation, and strengthened by fans for more intense convection during the hot summer period, the cowshed for lactating dairy cows (Table 1), which corresponds to the results found by [36,37,38], had average air temperatures from 24.9 ± 4.1 °C (LC NE) to 25.5 ± 4.3 °C (LC SW), with maximum temperatures t_max_ = 32.1 °C (LC NE) and t_max_ = 32.9 °C (LC SW). Slightly higher average temperatures in the LC SW part are caused by higher afternoon radiation from the west side of the cowshed. A similar effect was observed in LC SW for a slightly longer period, during which the values of THI ≥ 70 and THI ≥ 72 were exceeded (Table 3). Critical THI ≥ 78 values [26,27] were not exceeded during the measurements in the cowshed for lactating cows. The attenuation of AC_1d_ = 0.73 (LC SW) and AC_1d_ = 0.72 (LC NE) (Table 4) were favorable due to the relatively well-designed roof structure and a relatively high ceiling.

A similar situation as in the cowshed for lactating cows in terms of the thermal–humidity microclimate was also in the maternity cowshed. This is due to the similarly designed construction of the cowshed. The average air temperature during the hot summer period (Table 1) was 25.0 ± 5.5 °C, with the highest temperature t_max_ = 34.3 °C, which corresponds approximately to the maximum outdoor air temperature [67]. The relative humidity in the maternity cowshed 51.1 ± 18.3% was at a similar level as the relative humidity of the outdoor air, and the average value THI = 70.8 ± 4.8% and the maximum THI_max_ = 79.4 for the whole hot summer period corresponded to these values (Table 1). The time for which THI values were ≥70 and THI values were ≥72 (Table 3) was shorter than in the cowshed for lactating cows and even shorter than the outdoor air values. For the assessment, according to [64,65,66], the attenuation (AC_1d_ = 0.89) in maternity cowshed (Table 4) was small and slightly worse than in the cowshed for lactating cows.

As mentioned above, the worst situation during the hot summer period was in the calf housing facilities, the calf hutch, and the calf shelter. Exceedance of critical and, according to [26,27], dangerous THI values ≥ 78 during the measurement was 10.8 h in the calf hutch and 11.5 h in the calf shelter, which are values approaching 12 h. This was also reflected in the evaluation and modeling of the hot summer day (Table 4), when no attenuation was found, but on the contrary, there was an increase in the attenuation coefficient (AC) in both cases. The large fluctuations of the indoor temperature A_i_ are caused by a large increase in the indoor temperature during the day, which is due to solar radiation, and a large decrease in the indoor temperature at night, which is due to the low heat storage and negligible thermal insulation of the housing constructions, which also correspond to findings in [42,43,44,45]. In the calf hutch, the value of the attenuation coefficient was AC_1d_ = 1.65 and the time shift of the temperature maximum was Ψ_1d_ = −5.45 h, while in the calf shelter the attenuation coefficient was AC_1d_ = 1.57 and the time shift of the temperature maximum was Ψ_1d_ = −4.0 h. Negative values of Ψ_1d_ mean that there was no shift. On the contrary, due to the rapid heating caused by solar radiation, the indoor maximum temperature rose by several hours before the outdoor temperature maximum did.

The issue of the housing of milking cows outside the lactation period (dry cows) is not paid much attention in the literature. The old-but-modernized cowshed for dry cows, with its microclimate parameters in the hot summer period (Table 1), did not differ much from the outdoor conditions or from the maternity cowshed and cowshed for lactating cows. Due to less intensive ventilation in the afternoon, the air temperature inside the cowshed rose, but due to the more massive construction, there was a temperature attenuation of AC_1d_ = 0.7 (Table 3).

A relative humidity of 54.4 ± 13.9% was in the cowshed for dry cows for the whole hot summer period. The average value of THI = 71.8 ± 4.5% and the maximum THI_max_ = 79.1 corresponded to this (Table 1). These results are slightly higher than in the cowshed for lactating cows and the maternity cowshed. The critical and dangerous [26,27,42] value of THI ≥ 78 in the cowshed for dry cows was not exceeded, but THI ≥ 72 was for 13 h, which causes stress [11,26,27,42]. The access of dry cows to the open-roofed feeding area and the spacious modernized barn with plenty of space for rest, however, allows dry cows to move freely and choose a place to stay for a good rest, improving concerns about animal welfare.

## 5. Conclusions

This study has provided some insight into the influence of external temperature conditions on the thermal–humidity parameters of the internal environment on dairy farms in the summer. Climate change in recent years has also brought ever higher summer temperatures in Central European countries. High temperatures can have a serious and negative effect on the milk yield of dairy cattle. Modern dairy farms usually have a large capacity, which requires a solution for the suitable housing and construction of buildings for all categories and groups of animals on these farms. New high-capacity barns used for lactating dairy cows usually allow for good ventilation and moisture removal from the barn, but they should also provide good protection for cows from excessive solar radiation and reduce the risk of heat stress. This is also related to the choice of a suitable position of the building and the height and construction of the roof.

The results of this study showed that older brick cowsheds, used after reconstruction and modernization in many cases, for housing certain groups of cows, such as dry cows, or before and after calving, have better heat storage. This is reflected in a good attenuation of indoor air temperatures and a shift of the highest temperatures by several hours. Modern milking equipment installed in separate milking parlors provides the prerequisites for better milking hygiene, but sufficient ventilation must also be ensured, especially due to the large production of water vapor, which together with higher air temperature causes a high temperature–humidity index and thermal stress during milking. This research has shown that great attention should be paid to calf housing. During high summer temperatures, individual calf hutches or group calf shelters do not provide good protection for calves from heat stress. Calves are exposed to high temperatures for a long time during the day and suffer from great heat stress. It is necessary to focus on improving the structure, use suitable shading, or look for a more suitable solution for housing calves.

## Figures and Tables

**Table 1:** Results of measurement and statistical comparison of parameters of thermal–humidity environment of outdoor air and thermal–humidity environment of indoor air in all investigated buildings calculated for measured 4 days of the hot summer period (HSP). Different superscript letters (^a, b, c^) are a sign of a significant difference (*ANOVA*; *Tukey’s HSD test*; *p* ≤ 0.05) between the measured values in the individual columns. Identical superscript letters for numbers in a column indicate that there is no statistically significant difference between measured values in compared objects.

Measured Object	t ± SD °C	RH ± SD %	THI ± SD	t_max_ °C	THI_max_
External	24.8 ± 5.6 ^a^	51.0 ± 18.5 ^a,b,c^	70.5 ± 5.6 ^a^	34.9	79.0
MP	24.3 ± 2.9 ^a^	62.3 ± 13.5	71.7 ± 3.4 ^a^	29.6	78.4
LC SW	25.5 ± 4.1 ^a^	50.6 ± 19.0 ^a,b,c^	71.7 ± 3.3 ^a^	32.9	76.7
LC NE	24.9 ± 4.3 ^a^	51.4 ± 14.8 ^a,b,c^	71.0 ± 4.3 ^a^	32.1	77.7
MC	25.0 ± 5.5 ^a^	51.1 ± 18.3 ^a,b,c^	70.8 ± 4.8 ^a^	34.3	79.4
CaS	29.5 ± 10.2 ^b^	49.0 ± 27.1 ^a,b^	74.8 ± 9.4 ^b^	46.9	89.4
CaH	30.3 ± 11.9 ^b^	47.7 ± 29.2 ^a,b^	74.9 ± 11.3 ^b^	48.0	90.1
DC	25.2 ± 4.4 ^a^	54.4 ± 13.9 ^a,c^	71.8 ± 4.5 ^a^	33.2	79.1

SD—standard deviation. MP—milking parlor; LC SW—southwest cowshed for lactating cows; LC NE—northeast cowshed for lactating cows; MC—maternity cowshed; CaS—calf shelter; CaH—calf hutch; DC—cowshed for dry cows.

**Table 2:** Results of measurement and statistical comparison of parameters of thermal–humidity environment of outdoor air and thermal–humidity environment of indoor air in all investigated buildings during the hot summer day. All values in the individual columns are compared. Different superscript letters (^a,b,c,d^) are a sign of a significant difference (*ANOVA*; *Tukey’s HSD test*; *p* ≤ 0.05) between measured values in the individual columns. Identical superscript letters for numbers in a column indicate that there is no statistically significant difference between measured values in compared objects.

Measured Object	t_1d_ ± SD °C	RH_1d_ ± SD %	t_1d max_
External	25.1 ± 6.6 ^a^	51.4 ± 22.2 ^a,b,c,d^	34.9
MP	25.0 ± 3.3 ^a^	61.3 ± 17.2	29.6
LC SW	25.8 ± 5.0 ^a^	49.8 ± 22.6 ^a,b,c^	32.8
LC NE	25.1 ± 5.1 ^a^	51.7 ± 18.2 ^a,b,c^	31.3
MC	25.1 ± 6.3 ^a^	51.8 ± 21.7 ^a,b,c^	33.5
CaS	29.8 ± 11.5 ^b^	51.3 ± 30.8 ^a,b^	46.6
CaH	29.9 ± 12.9 ^b^	49.9 ± 32.9 ^a,b^	46.4
DC	25.3 ± 5.0 ^a^	54.2 ± 16.2 ^a,c^	31.8

SD—standard deviation. MP—milking parlor; LC SW—southwest cowshed for lactating cows; LC NE—northeast cowshed for lactating cows; MC—maternity cowshed; CaS—calf shelter; CaH—calf hutch; DC—cowshed for dry cows.

**Table 3:** Results of measurements and statistical comparison of THI values of the external thermal–humidity environment of outdoor air and all studied housing objects during the hot summer day. Different superscript letters (^a,b^) are a sign of a significant difference (*ANOVA*; *Tukey’s HSD test*; *p* ≤ 0.05) between calculated THI_1d_ values of measured objects. Identical superscript letters for numbers in a column indicate that there is no statistically significant difference between measured values in compared objects.

Measured Object	THI_1d_ ± SD	THI_1d_ ≥ 70		THI_1d_ ≥ 72		THI_1d_ ≥ 78		THI_1d max_
Time	-	h	%	h	%	h	%	-
External	70.5 ± 6.7 ^a^	15.0	62.5	13.5	56.3	1.8	7.3	78.6
MP	72.5 ± 3.7 ^a^	18.8	78.1	16.8	69.8	0.8	3.1	78.4
LC SW	71.6 ± 4.1 ^a^	16.5	68.8	14.3	59.4	0	0	76.2
LC NE	71.1 ± 5.1 ^a^	16.0	66.7	13.5	56.3	0	0	76.7
MC	70.6 ± 6.3 ^a^	14.5	60.4	12.3	51.0	0	0	77.9
CaS	74.7 ± 10.8 ^b^	14.5	60.4	13.8	57.3	11.5	47.9	89.3
CaH	73.9 ± 12.4 ^b^	14.3	59.4	13.8	57.3	10.8	44.8	89.5
DC	71.8 ± 5.1 ^a^	16.0	66.7	13.0	54.2	0	0	77.8

SD—standard deviation. MP—milking parlor; LC SW—southwest cowshed for lactating cows; LC NE—northeast cowshed for lactating cows; MC—maternity cowshed; CaS—calf shelter; CaH—calf hutch; DC—cowshed for dry cows.

**Table 4:** The results of measured average air temperatures t_1d_ ± SD °C, attenuation coefficient AC_1d_, time shift of temperature maximum Ψ_1d_, model curve equations expressing the course of air temperature during 24 h of the hot summer day and average temperatures t_1dm_ ± SD °C calculated from model waveforms. Identical superscript letters (^a^) are a sign that there is not a significant statistical difference (*ANOVA*; *Tukey’s HSD test*; *p* ≤ 0.05) between measured value t_1d_ ± SD °C and model value t_1dm_ ± SD °C in the same line. Identical superscript letters for numbers in a line indicate that there is no statistically significant difference between measured and modeled values.

Measured Object	t_1d_ ± SD °C	AC_1d_	Ψ_1d_ h	t_1dm_ ± SD °C	Model Equation of Temperature Course
External	25.1 ± 6.6 ^a^	-	-	24.3 ± 6.9 ^a^	t_em_ = 34.3–9.8 [1–sin (15 τ–153)]
MP	25.0 ± 3.3 ^a^	0.55	0.75	24.1 ± 3.8 ^a^	t_im_ = 29.6–5.5 [1–sin (15 τ–165)]
LC SW	25.8 ± 5.0 ^a^	0.73	−0.5	25.3 ± 5.1 ^a^	t_im_ = 32.8–7.4 [1–sin (15 τ–163)]
LC NE	25.1 ± 5.1 ^a^	0.72	0	23.9 ± 5.0 ^a^	t_im_ = 31.2–7.2 [1–sin (15 τ–160)]
MC	25.1 ± 6.3 ^a^	0.89	0	24.3 ± 6.1 ^a^	t_im_ = 33.2–8.8 [1–sin (15 τ–155)]
CaS	29.8 ± 11.5 ^a^	1.57	−4.0	29.5 ± 10.5 ^a^	t_im_ = 44.8–14.9 [1–sin (15 τ–135)]
CaH	29.9 ± 12.9 ^a^	1.65	−5.45	29.1 ± 11.9 ^a^	t_im_ = 46.4–16.7 [1–sin (15 τ–131)]
DC	25.3 ± 5.0 ^a^	0.7	−1.0	24.6 ± 4.8 ^a^	t_im_ = 31.6–6.9 [1–sin (15 τ–160)]

SD—standard deviation. MP—milking parlor; LC SW—southwest cowshed for lactating cows; LC NE—northeast cowshed for lactating cows; MC—maternity cowshed; CaS—calf shelter; CaH—calf hutch; DC—cowshed for dry cows.

## Data Availability

Not applicable.
